# Towards an Optimal KELM Using the PSO-BOA Optimization Strategy with Applications in Data Classification

**DOI:** 10.3390/biomimetics8030306

**Published:** 2023-07-12

**Authors:** Yinggao Yue, Li Cao, Haishao Chen, Yaodan Chen, Zhonggen Su

**Affiliations:** 1 School of Intelligent Manufacturing and Electronic Engineering, Wenzhou University of Technology, Wenzhou 325035, China; yueyinggao2006@163.com (Y.Y.); caoli198723@163.com (L.C.); 00195124@wzut.edu.cn (H.C.); elevenward@sina.cn (Y.C.); 2 Intelligent Information Systems Institute, Wenzhou University, Wenzhou 325035, China; 3 Taishun Research Institute, Wenzhou University of Technology, Wenzhou 325035, China

**Keywords:** kernel extreme learning machine, butterfly optimization algorithm, particle swarm optimization, parameter optimization, generalization ability

## Abstract

The features of the kernel extreme learning machine—efficient processing, improved performance, and less human parameter setting—have allowed it to be effectively used to batch multi-label classification tasks. These classic classification algorithms must at present contend with accuracy and space–time issues as a result of the vast and quick, multi-label, and concept drift features of the developing data streams in the practical application sector. The KELM training procedure still has a difficulty in that it has to be repeated numerous times independently in order to maximize the model’s generalization performance or the number of nodes in the hidden layer. In this paper, a kernel extreme learning machine multi-label data classification method based on the butterfly algorithm optimized by particle swarm optimization is proposed. The proposed algorithm, which fully accounts for the optimization of the model generalization ability and the number of hidden layer nodes, can train multiple KELM hidden layer networks at once while maintaining the algorithm’s current time complexity and avoiding a significant number of repeated calculations. The simulation results demonstrate that, in comparison to the PSO-KELM, BBA-KELM, and BOA-KELM algorithms, the PSOBOA-KELM algorithm proposed in this paper can more effectively search the kernel extreme learning machine parameters and more effectively balance the global and local performance, resulting in a KELM prediction model with a higher prediction accuracy.

## 1. Introduction

Data classification is one of the most important research hotspots in the field of high-tech at present. It uses certain features to discriminate or classify a group of objects. The information involved in data classification often has the characteristics of high dimensions, many influencing factors, and complex relationships [1]. It is often difficult to effectively determine its laws by human thinking alone, and it needs to be completed through certain mathematical methods with the help of computers. How to discover more and more valuable, associated information from this complex data information, find its internal laws, and establish a model that can better reflect the actual characteristics of the research object, be easily integrated with prior knowledge, and be adaptable to large-scale data-processing requirements are gradually becoming the focus of current data classification [2]. Multi-label classification, or MLC, or the study of a thing according to many class label ideas, has become particularly significant in order to address the inadequacies of conventional single-label classification. The multi-label data flow exhibits the features of vast speed and idea drift in the sphere of practical application, making the conventional multi-label classification algorithm unable to directly address such issues. Designing a reliable multi-label data flow classification approach has therefore emerged as a crucial and difficult challenge due to the need to process these new data fast while having limited time and memory as well as to adjust the concept drift in the data flow environment [3].

In engineering applications, learning for data classification is very desirable. Traditional learning techniques, such as artificial neural networks (ANN) and support vector machines (SVM), appear helpless in the face of the twin needs of quicker training speed and greater learning accuracy [4,5]. Huang et al.’s extreme learning machine (ELM) is a single-layer feedforward network learning technique (single-hidden layer feedforward neural networks, SLFNs) that does not need changing the hidden layer neuron network settings. It offers quick training and effective learning outcomes, which are important characteristics. Due to this, the distributed extreme learning machine (D-ELM) avoids reading all samples into memory at once by partitioning matrix operations, but it also resolves the issue of memory shortage while training vast amounts of sample data [6,7]. Every time they run, they only train an ELM network with a certain amount of hidden layer nodes. Similar to cross-validation, they do not take into account the generalization capacity under various training and test set divisions. They want to improve their generalization skills and accuracy. Numerous runs of the solution to must be performed. When comparing the generalization capacity or the network performance of various hidden layer node numbers, it is impossible to ensure that the hidden layer network parameters are consistent, making it difficult to accurately and intuitively learn their impact on the model [8]. This is due to the random generation of hidden layer network parameters.

### 1.1. Problem Description and Research Motivation

In recent years, many scholars have applied neural network-based algorithms to data classification research, such as BP neural network, discrete Hopfield network, support vector machine, self-organizing network, fuzzy neural network, and generalized neural network, and achieved many results. Since most neural networks use the gradient descent method, there are often shortcomings, such as slow training speed, easy to fall into local minimum, and learning rate sensitivity. Therefore, it is necessary to explore a fast-training speed, accurate optimal solution, and good generalization The performance of the training algorithm is the main goal of improving the performance of the neural network.

The KELM model relies on both feature selection and parameter optimization, and these two processes are complementary and cooperative. To avoid overfitting and lower the computational cost of the training model, feature selection chooses the most pertinent and discriminative feature subset from the original feature space and eliminates redundant and unimportant features [9]. A proper parameter setup can significantly enhance the KELM model’s classification performance and yield superior classification outcomes. These two elements are taken into account during the design and are optimized simultaneously to increase the KELM model’s capacity for generalization [10].

Due to its strong search capabilities, the meta-heuristic algorithm has received considerable attention recently. Numerous study findings have established that this algorithm is superior to conventional approaches for solving optimization issues [11]. Researchers working on this project have to date suggested a few optimization strategies for parameter and feature selection. For instance, Alcin et al. introduced the genetic algorithm (GA) to KELM model in 2014 after using the GA method to improve the sparse output weight vector of the KELM model. The kernel KELM model’s parameters were optimized using the particle swarm optimization (PSO) technique by Bin Li et al. in the same year [12,13].

The primary issues with KELM’s categorization model are:

The selection of pertinent parameters is a significant issue for both the KELM study model and the SVM model, although there are not many studies on this topic [14].

It is common to conduct feature selection and parameter optimization independently. In essence, the two cooperate and support one another. The best model cannot be assured if they are optimized individually [15].

A novel classification method for kernel extreme learning machines (PSOBOA-KELM) based on the modified PSO butterfly optimization algorithm is suggested in order to solve the aforementioned issues. This technique concurrently performs parameter optimization of the kernel ELM model and feature selection based on the enhanced butterfly algorithm (PSOBOA) to increase KELM’s generalization performance [16,17]. The particle swarm optimization algorithm is introduced at the same time to improve the GSA algorithm’s performance in optimization by addressing the latter stages of iteration’s slow convergence speed and weak local search ability, and the chaos control strategy is intended to broaden the group’s diversity. The PSOBOA-KELM technique suggested in this work simultaneously maximizes the number of hidden layer nodes and generalization ability. It saves a vast amount of time by dividing training into segments based on samples. Thus, potential solutions should be investigated.

### 1.2. Contribution

Compared with the traditional method, the butterfly optimization algorithm optimized by particle swarm optimization proposed in this paper comprehensively considers issues, such as improving the classification accuracy of the algorithm and the generalization performance of KELM. The main contributions of this paper are as follows:

1. Characterize the swarm intelligence optimization algorithm/butterfly optimization algorithm and classify the current data classification methods.
2. Propose a novel data classification method of kernel extreme learning machine based on the butterfly optimization algorithm optimized by particle swarm optimization (PSOBOA-ELM).
3. Provide extensive simulation results to demonstrate the use and efficiency of the proposed data classification method.
4. Evaluate the performance of the proposed algorithms by comparing them with the data classification methods of other algorithms.

The remainder of this paper is organized as follows: Section 2 discusses the related work. Section 3 describes the basic principles of kernel extreme learning machine. Section 4 describes the principles of the butterfly optimization algorithm optimized using particle swarm optimization. Section 5 describes the implement steps of the proposed algorithm design idea of the kernel extreme learning machine method based on the butterfly optimization algorithm optimized by particle swarm optimization. Section 6 provides the parameters and simulation results that validate the performance of the proposed algorithm. Section 7 concludes the paper.

## 2. Related Work

Data categorization is frequently employed as the fundamental processing technique in contemporary intelligent data processing. Machine learning is a powerful tool for achieving the objective of data processing since it makes use of data sets to create classification models with great generalization capabilities.

Support vector machines are at present being used by some academics to handle multi-instance learning challenges. The conventional SVM is particularly sensitive to noisy points and singular points in the sample. The CA-SVM based sentiment analysis model that Cyril et al. suggested uses automatic learning to read Twitter datasets, analyze them, and extract features to provide a list of phrases [18]. More characteristics in the input electrocardiosignal (ECG) signal were classified using the SVM model and weighted kernel function approach by Varatharajan et al. At present, the existing multi-label classification methods mainly include: batch processing methods and online learning methods. Among them, the batch processing method defaults to the one-time arrival of each training and testing data set, and uses problem transformation and algorithm self-adaptation to solve multi-label classification problems based on all existing information. The extreme learning machine (ELM) proposed by Huang and its improved algorithm have the characteristics of high speed and high efficiency, avoiding the cumbersome iterative learning process [19]. The random setting of learning parameters caused by the iterative learning of the traditional feed-forward neural network can easily encounter problems, such as the local minima, while improving the algorithm can further improve the classification accuracy. Therefore, related research based on (kernel) extreme learning machines has been widely applied to multi-label classification problems, and a series of results has been achieved [20]. However, due to the characteristics of massive and fast data streams emerging in the practical application field, it is difficult to obtain them all at once. At the same time, when new data arrives, these batch-processing algorithms continue to retrain new data and discard old models, resulting in a large loss of effective historical data [21]. Therefore, learning models that can handle data stream environments are receiving more and more attention.

Ensemble techniques have been one of the most significant advancements in machine learning over the past ten years. In actuality, the kernel function is utilized as a crucial theoretical tool in the data preprocessing since the data in the data set are linearly indistinguishable, making it necessary to build an appropriate data classification procedure. The goal of the kernel function is to discover the classification hyperplane of the low-dimensional, indistinguishable data in the new high-dimensional space by nonlinear transformation, allowing for the separation of the data. The construction and parameter selection of the kernel function are at present its key areas of emphasis. To address the multi-class unbalanced data classification problem, Zhang et al. suggested a support vector machine (SVM) technique based on a proportional kernel and proposed a scaling kernel function, which employs weighting factors to compute its parameters. The issue of skewed distribution-induced classifier performance reduction has a high degree of generalizability [22]. To discriminate between various kinds of ground objects, Chen et al. presented a novel hybrid kernel function SVM point cloud classification technique, and they created a Gaussian and polynomial hybrid kernel function to increase the classification accuracy [23]. Xie et al. calculated the similarity of samples with several unknown attributes using the characteristics of kernel functions. To compute the kernel function and solve additive kernel singular values, an effective technique was also shown [24]. Zhang et al. developed a novel conformal function to scale the kernel matrix of ODM in order to increase the separability of the training data in the feature space. They also presented a kernel modified ODM kernel function (KMODM) to remove the unbalanced data classification approach [25]. When tackling small sample, nonlinear, and high dimensional problems, it demonstrates several distinct benefits and may be successfully applied to various machine learning disciplines.

Although it has been demonstrated that the Ada Boost approach, which uses a neural network as the basic classifier, has a high generalization performance, training is still not without its challenges. Diversity has undergone extensive research as a crucial component in the generalization performance of classifiers, and certain approaches to assess diversity have also been presented. In order to overcome the limitations of fixed representations, Deng et al. used deep learning to perform large-scale task-driven feature learning from big data. They also demonstrated its utility in image classification, high-frequency financial data prediction, and brain MRI And how well these three duties may be divided [26]. Saritas et al. assessed the classification performance of a Bayesian classifier and an artificial neural network applied to nine inputs and one output and compared the findings [27]. By fusing morphological neurons with perceptrons, Gerardo et al. suggested two novel hybrid neural architectures. They then evaluated them using 25 low-dimensional standard data sets and a large data set. The suggested approach achieved improved accuracy while using fewer learning parameters [28]. In contrast to conventional techniques and other state-of-the-art techniques, Wu et al. used a convolutional recurrent neural network (CRNN) to learn more discriminative features for hyperspectral data classification, using recurrent layers to further extract spectral context information from features generated by convolutional layers. The suggested technique offers improved classification performance for hyperspectral data classification when compared to deep learning methods [29].

At present, some achievements have used the sliding window technology to apply extreme learning machines to solve the multi-label classification of data streams, but this method does not consider the problem of class label correlation between multiple labels and concept drift in the data stream environment. On the other hand, some researchers pointed out that, when dealing with data streams, it is necessary to consider the model to make accurate predictions under limited time and memory and include solutions to the problem of concept drift. These requirements pose more challenges to the classification of multi-label data streams. Most of the multi-label classification algorithms in the data flow environment use problem transformation to convert classification into a series of stable learning tasks. Although this method can be applied to a certain extent, it ignores the correlation between labels. At the same time, it does not take into account the high-speed and changeable characteristics of the newly arrived data, and the implicit concept drift problem is also difficult to solve by the problem transformation method. One of the challenging issues in data mining is data categorization, a topic that has attracted considerable attention from both domestic and international scholars studying artificial intelligence. It is required to fundamentally optimize the imbalanced data in order to address the issue of unbalanced data categorization. At present, the outcomes of academic research are improving yearly. Nearly 900 scholarly publications on unbalanced data categorization were published between May 2018 and 2022, a significant increase over the preceding ten years.

## 3. Kernel Extreme Learning Machine

The extreme learning machine consists of three parts: input layer, hidden layer, and output layer. For a given training sample, continuously optimize the input weights and bias values between the connected input layer and the hidden layer, and maintain them unchanged during the training process. Assume a training sample set of $\left\{x_{i},c_{i}\right\},i=1,2,$…, *N* is given, where *x_i_* is the input value of the training sample and *c_i_* is the corresponding output value. Let the limit learning machine have h hidden layer nodes, the network output is *f*, and *g*(*) is the activation function; then, the input and output model of the limit learning machine can be expressed by Formula (1) [30].

(1) $$f(x_{j})=\sum_{i=1}^{h}\beta_{i}g(\omega_{i}\times x_{j}+b_{i}),j=1,2,\ldots N$$

In the formula, the output weight of the input node and the *i*-th hidden layer node are represented by $\beta_{i}$. The input weight of the *i*-th hidden node and the input node are represented by $\omega_{i}$. The offset value of the *i*-th hidden node is represented by *b_i_*.

(2) $$\begin{array}{l} H={\left[\begin{array}{l} g(\omega_{1}\times x_{1}{+b}_{1})\;\cdot \cdot \cdot \;g(\omega_{L}\times x_{1}{+b}_{L})\\ \begin{array}{ccc} g(\omega_{1}\times x_{2}{+b}_{1}) & \ldots & g(\omega_{L}\times x_{2}{+b}_{L})\\ \vdots & \ddots & \vdots \\ g(\omega_{1}\times x_{N}{+b}_{1}) & \cdots & g(\omega_{1}\times x_{N}{+b}_{1}) \end{array}\; \end{array}\right]}_{N\times L}\\ \beta ={\left[\begin{array}{l} \beta_{1}\\ \beta_{2}\\ \vdots \\ \beta_{L} \end{array}\right]}_{L\times 1},T={\left[\begin{array}{l} c_{1}\\ c_{2}\\ \vdots \\ c_{L} \end{array}\right]}_{L\times 1} \end{array}$$

The output weight can be expressed by Formula (3).

(3) $$\hat{\beta }=H^{*}T$$

In the equation, $H^{*}$ is the inverse of matrix *H*.

Replace the hidden layer in the extreme learning machine with the idea of kernel function mapping in the support vector machine. Then, the kernel extreme learning machine can be expressed by Formula (4) [31].

(4) $$\begin{array}{l} \min P_{ELM}=\frac{1}{2}{\left\|\beta \right\|}^{2}+\frac{C}{2}\sum_{i=1}^{N}\xi_{i}^{2}\\ s.t.h(x_{i})\beta =c_{i}-\xi_{i} \end{array}$$

Therefore, the input and output model of the kernel extreme learning machine is as shown in Formula (5).

(5) $$f(x)=h(x)\beta =h(x)H^{T}{\left(\frac{I}{C}+HH^{T}\right)}^{-1}T$$

Define the extreme learning machine kernel matrix as Formula (6) [32].

(6) $$\begin{array}{l} \Omega_{ELM}=HH^{T}\\ \Omega_{ELMi,j}=h(x_{i})•h(x_{j})=K(x_{i},x_{j}) \end{array}$$

The corresponding input–output model can be expressed as shown in Formula (7).

(7) $$f(x)={\left[\begin{array}{l} K(x,x_{1})\\ K(x,x_{2})\\ \vdots \\ K(x,x_{N}) \end{array}\right]}^{T}{\left(\frac{I}{C}+\Omega_{ELM}\right)}^{-1}T$$

The feature mapping h(x) of the hidden layer is unknown in kernel limit learning machines, but it is usually calculated using the kernel K(μ,ν) ($K(\mu ,\nu )=\exp(-\gamma {\left\|\mu -\nu \right\|}^{2})$) to reduce the impact of poor classification results caused by the unreasonable setting of the number of hidden layer nodes (the dimension of feature space).

As a result, kernel ELM has the benefit of effective ELM SVM classification. The number of hidden layer nodes need not be predetermined because KELM determines the hidden layer mapping kernel function in the form of an inner product by introducing a kernel function. As a result, the generalization of the KELM-based electric load forecasting model results in significantly increased capacity and stability.

## 4. Butterfly Optimization Algorithm Optimized by PSO

### 4.1. Butterfly Optimization Algorithm

A novel meta-heuristic algorithm called the butterfly optimization algorithm (BOA) mimics the foraging and courting behavior of butterflies. It has a high resilience and global convergence ability while solving complicated functions [33,34]. The BOA algorithm has two crucial inputs: switching probability and scent. The switching probability determines the likelihood that the butterfly will select one of two movement modes, either global or local, and the smell stands in for the quality of the particular butterfly’s current position [35]. The butterfly colony is first dispersed at random in the solution space, and the butterflies that have a strong scent draw other individual butterflies to them. The aim optimization is accomplished by consistently updating the butterfly colony’s location [36]. Each butterfly in the butterfly optimization algorithm has a distinct scent and perception ability, and the strength of smell perception varies between individuals. Formula (8) illustrates how strongly other butterflies can smell a person among them:

*f*(*x*) = *cI^a^* (8)

Among them, *f*(*x*) represents the odor intensity function; *c* represents the sensory shape coefficient; *I* represents the stimulus intensity, that is, the fitness value of the function; and *a* represents the intensity coefficient, and the value is in [0, 1].

The sensory shape coefficient *c* can theoretically take any value within [0, ∞), and its calculation is shown in Formula (9):

*c_t_*_+1_ = *c_t_* + [0.025/(*c_t_*·*T_max_*)] (9)

Among them, the initial value of *c* is 0.01, and *T_max_* is the maximum number of iterations of the algorithm. The BOA algorithm determines the global search and local search of the algorithm according to the switching probability *p*, and the position update formula is shown in Formula (10):

(10) $$x_{i}{}^{t+1}=\{\begin{array}{c} x_{i}{}^{t}+(r^{2}\cdot g^{*}-x_{i}{}^{t})\cdot f_{i},p<rand\\ x_{i}{}^{t}+(r^{2}\cdot x_{j}{}^{t}-x_{k}{}^{t})\cdot f,p\geq rand \end{array}$$

Among them, g* is the best position of all butterflies in the current iteration; *x^t^_j_* and *x^t^_k_* represent the spatial positions of the *j*-th butterfly and the *k*-th butterfly in the *t*-th iteration, respectively; the value of *r* is a random number of [0,1] number; and *f_i_* is the fitness value of the *i*-th butterfly.

### 4.2. Particle Swarm Optimization Algorithm

A swarm intelligence optimization system called particle swarm mimics how birds fly while looking for food in a multidimensional search environment. Particle position and velocity are the two key aspects of the PSO method optimization [37,38]. Each one of them is referred to as a particle, and each particle’s initial position and velocity in the search space are initialized at random [39]. The particles’ positions and velocities are updated in accordance with Formulas (11) and (12):

(11) $$v_{i}{}^{t+1}=\omega \cdot v_{i}{}^{t}+c_{1}\cdot rand_{1}\cdot (p_{best}-x_{i}{}^{t})+c_{2}\cdot rand_{2}\cdot (g_{best}-x_{i}{}^{t})$$

(12) $$x_{i}{}^{t+1}=x_{i}{}^{t}+v_{i}{}^{t+1}$$

Among them, *v^t^_i_* and *v_i_^t^*^+1^ represent the velocities of the *i*-th particle at the *t* and *t* + 1 iterations, respectively; and *p_best_* and *g_best_* represent the initial and global optimal positions of particles, respectively. Generally, the hyperparameter *c*_1_ = *c*_2_ = 2; *rand*_1_ and *rand*_2_ are random numbers of (0, 1); and *ω* represents the inertia weight coefficient.

### 4.3. Butterfly Optimization Algorithm Optimized by PSO (PSOBOA)

(1) Algorithm population initialization

Assume that, in the D-dimensional search space, the greedy strategy is used to generate a new race to generate the initial solution expression, which is shown in Formula (13):

(13) $$X_{i}=L_{b}+(U_{b}-L_{b})\cdot O$$

Among them, *X_i_* represents the spatial position of the *i*-th butterfly (*i* = 1, 2, 3,…, *N*) in the butterfly population, and *N* represents the number of initial solutions. *L_b_* and *U_b_* represent the upper and lower bounds of the search space, respectively; and *O* represents a matrix of random numbers with elements (0, 1).

(2) Algorithmic Global Search

The global search phase of butterfly optimization algorithm optimized by PSO (PSOBOA) can be expressed by Formulas (14) and (15):

(14) $$X_{i}{}^{t}=\omega \cdot X_{i}{}^{t-1}+(r^{2}\cdot g_{best}-\omega \cdot X_{i}{}^{t-1})\cdot f_{i}$$

(15) $$X_{i}{}^{t+1}=X_{i}{}^{t}+\omega \cdot V_{i}{}^{t}+C_{1}\cdot r_{1}\cdot (p_{best}-X_{i}{}^{t})+C_{2}\cdot r_{2}\cdot (g_{best}-X_{i}{}^{t})$$

Among them, *ω* represents the inertia weight coefficient, and *V^t^_i_* and *V_i_^t^*^+1^ represent the velocities of the *i*-th particle at time *t* and *t* + 1, respectively. The hyperparameter *C*_1_ = *C*_2_ = 2, and the values of *r*_1_ and *r*_2_ are random numbers (0, 1).

(3) Local search of algorithms

The local search stage of the PSOBOA algorithm can be represented by Equations (16) and (17):

(16) $$X_{i}{}^{t}=\omega \cdot X_{i}{}^{t-1}+(r^{2}\times X_{k}{}^{t-1}-\omega \cdot X_{j}{}^{t-1})\cdot f_{i}$$

(17) $$X_{i}{}^{t+1}=X_{i}{}^{t}+\omega \cdot V_{i}{}^{t}+C_{1}\cdot r_{1}\cdot (p_{best}-X_{i}{}^{t})+C_{2}\cdot r_{2}\cdot (g_{best}-X_{i}{}^{t})$$

Among them, *X_k_^t^*^−1^ and *X_J_^t^*^−1^ are the positions of the *k*-th and *j*-th butterflies randomly selected from the solution space of the *t* − 1 iteration, respectively; and *ω* represents the inertia weight coefficient. *C*_1_ = *C*_2_ = 2, *r*_1_, and *r*_2_ are random numbers with the values of (0, 1).

(4) Control strategy

Chaos theory has many applications in swarm intelligence optimization algorithm, such as chaos population initialization and chaos adjustment strategy of control parameters. Logistic mapping is a classical chaotic mapping method in chaos theory, and its expression is shown in Formula (18):

(18) $$z_{l+1}=\mu z_{l}(1-z_{l})$$

Among them, *l* represents the number of iterations of the chaotic map and *μ* is the chaotic parameter, and its value is in [0, 4]. The chaotic sequence of logistic mapping is (0, 1); when *μ* = 4, the mapping produces a chaotic phenomenon.

The Lyapunov index is an important index to distinguish the characteristics of chaos. The larger the maximum Lyapunov exponent of the chaotic map, the more obvious its chaotic characteristics and the higher the degree of chaos. The index expression is shown in Formula (19):

(19) $$\lambda =\underset{n\to \infty }{\lim}\frac{1}{n_{h}}\sum_{i=0}^{n_{h}-1}\ln\left|f^{\prime }(z_{i})\right|$$

where *λ* represents the Lyapunov exponent; $f^{\prime }(\cdot )$ represents the first derivative of the chaotic mapping function; and *n_h_* represents the number of iterations of the chaotic mapping.

The expression of the sensory shape coefficient *c* in the PSOBOA algorithm is shown in Formula (20):

(20) c(t)=4⋅c(t−1)⋅(1−c(t−1))

The inertia weight coefficient ω has a direct impact on the particle flight speed of the PSO algorithm and can adjust the global search and local search capabilities of the algorithm. In this paper, an adaptive adjustment strategy was adopted, as shown in Formula (21):

(21) $$\omega (t)=\omega_{\max}-\left((\omega_{\max}-\omega_{\min})\cdot T_{i}\right)/T_{\max}$$

Among them, *ω_max_* = 0.9, *ω_min_* = 0.2, and *T_max_* is the maximum number of iterations of the algorithm.

(5) Algorithm complexity analysis

Assuming that the number of populations of the algorithm is *N*, the dimension of the search space is *D*, and the maximum number of searches is *T_max_*, the complexity of PSOBOA includes: the initialization complexity of the population *O*(*ND*), the fitness value calculation complexity *O*(*ND*), the global and the position update complexity of local search *O*(*N*^2^log*N*), the fitness value sorting complexity of the algorithm *O*(*N*^2^), and the control parameter update complexity of the algorithm *O*(*ND*). Then, the complexity of PSOBOA algorithm is shown in Formula (22):

(22) $$O(HPSBA)=O(ND)+O(T_{\max})O(ND+N^{2}\log N+N^{2}+ND)$$

The algorithm time complexity of PSOBOA is shown in Formula (23):

(23) $$O(BOA)=O(ND)+O(T_{\max})O(N^{2}\log N+N+ND)$$

## 5. Data Classification of KELM Based on PSOBOA Algorithm

The regularization coefficient *C* and the kernel function parameter *S* of the kernel extreme learning machine were optimized using the particle swarm optimization butterfly technique, which raises the network’s classification recognition accuracy. We created the data categorization mathematical model after obtaining the optimal parameters. The following are the precise PSOBOA-KELM steps:

(1) Establish a classification database, divide it into training and testing sets, and normalize them.
(2) Train KELM using the training set as the input vector.
(3) The PSOBOA algorithm was used to optimize the regularization coefficient *C* and kernel function *S* in KELM. Select the optimal *C* and *S* and reconstruct KELM.
(4) Train the KELM algorithm optimized by PSOBOA again and compare the results.
(5) Determine whether the termination conditions are met. If satisfied, exit the loop and output the prediction result. Otherwise, it is recalculated.
(6) Input the test set into the optimized KELM and output the prediction results.

**Figure 1:**
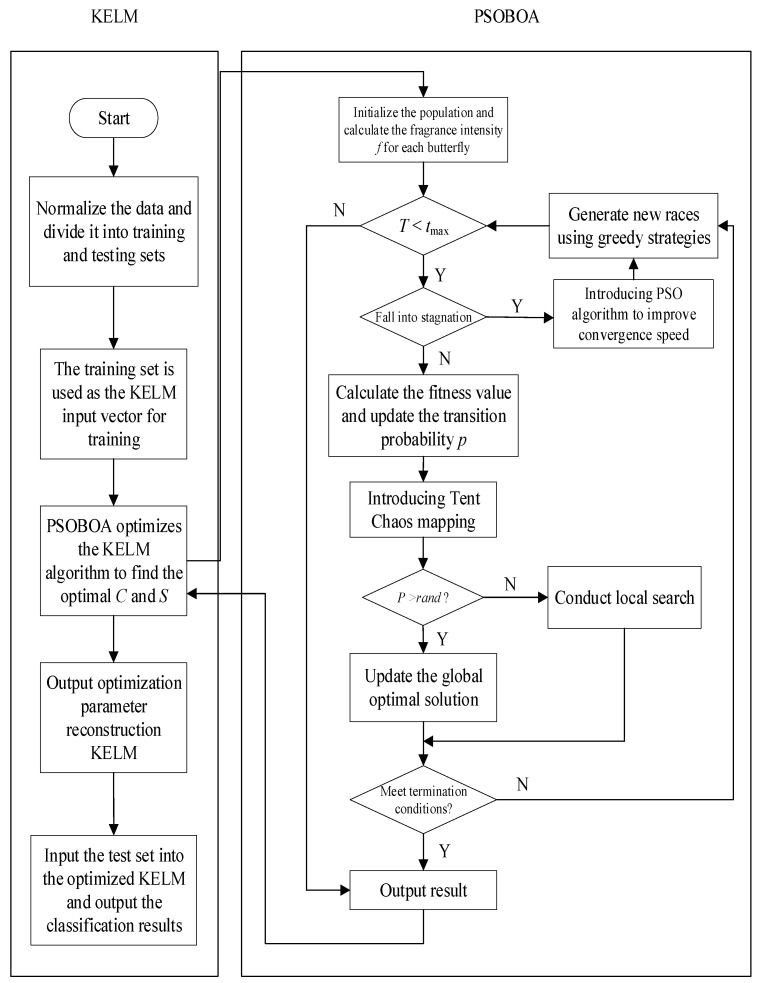
Flow chart of the PSOBOA-KELM algorithm.

According to the above steps, the flow chart of the PSOBOA-KELM algorithm is shown in Figure 1.

## 6. Algorithm Simulation and Result Analysis

### 6.1. Benchmark Function Test

In order to test the performance of the PSOBOA algorithm, eight test functions were used for testing, and it was compared with the particle swarm optimization algorithm (PSO), crow search algorithm (CSA), binary bat algorithm (BBA), and butterfly optimization algorithm (butterfly optimization algorithm, BOA). The improved PSOBOA algorithm was compared and analyzed. The eight test functions in CEC2017 were all evaluated as minimization problems, which were divided into multimodal test functions, mixed functions, and composite functions. The test functions are shown in Table 1. For fair comparison, the solution dimension of all test functions was 30, the population size was set to 30, the search space was all [−100, 100], all algorithms were run independently on each test function 30 times, and the maximum number of iterations for each run was for 100.

**Figure 2:**
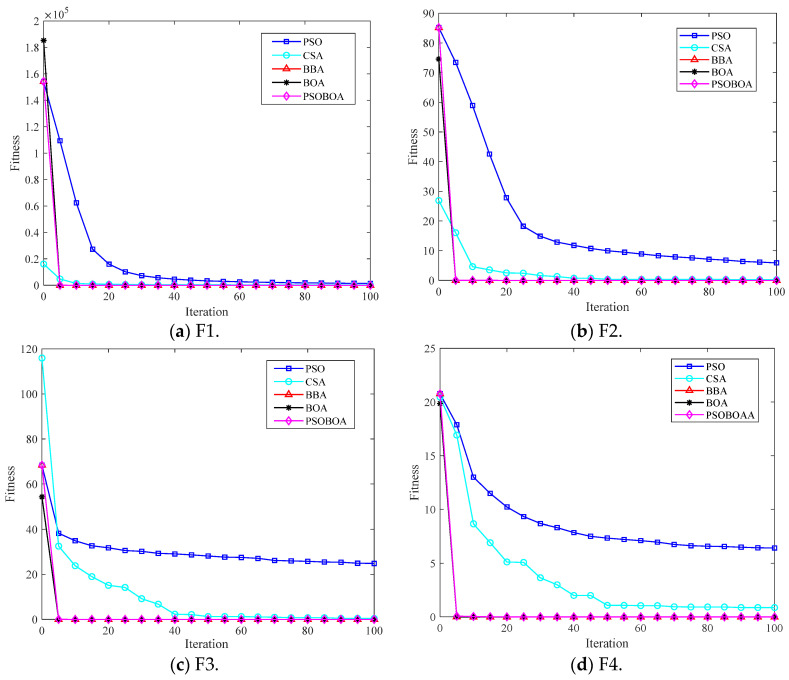

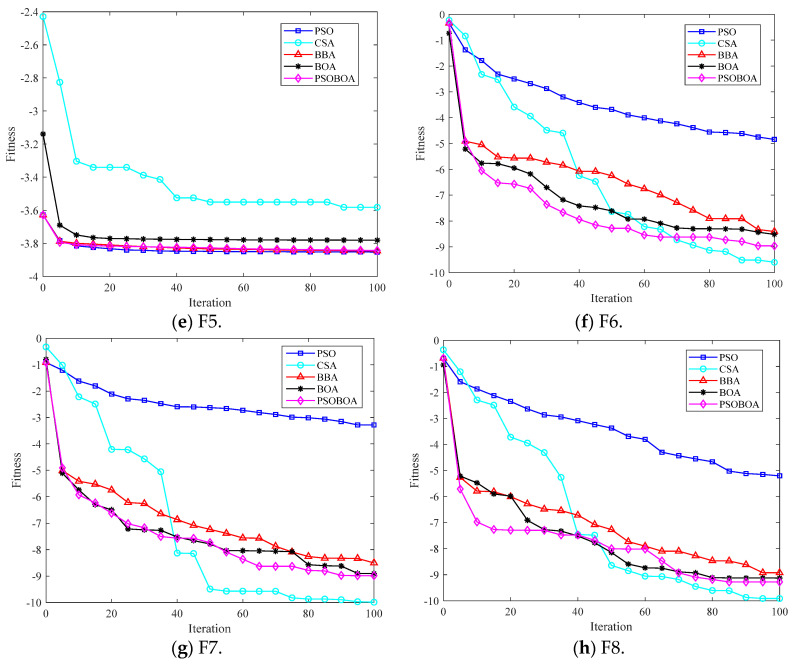
Comparison of the function iteration calculation.

In this paper, the results of PSO, CSA, BBA, BOA, and PSOBOA algorithms independently running 30 times on eight test functions were counted. The iterative calculation results of the test functions of the five algorithms are shown in Figure 2.

It can be seen from Figure 2 that, when solving the test function, the optimization results of the BBA, BOA, and PSOBOA algorithms are not much different, but they are all significantly better than the PSO algorithm and CSA algorithm. When solving the multi-peak test function, although the CSA algorithm achieves better results on the two test functions, according to the average ranking of the five algorithms on the multi-peak test function, the PSOBOA algorithm is better than the other four algorithms, the convergence speed is faster. When solving the mixed function, the PSOBOA algorithm achieved the best results on the test functions. When solving the composite function, the optimization effect of the PSOBOA algorithm is not significant compared with the BBA algorithm and BOA algorithm, but it can be seen from the comprehensive mean and standard deviation that the PSOBOA algorithm has high optimization and stable results.

At the same time, it can be seen from the experimental data in Figure 2 that for F3, F4, F6, and F8, PSOBOA has the strongest optimization performance, which is obviously better than PSO, CSA, BOA, and BBA, and F1, F2, F3, and F4 can directly find the optimal value of 0. For F7, the optimization performance of PSOBOA and BOA is almost the same, the average of optimization is slightly better than BBA, and the effect of PSO is the worst. For F6, the optimization performance of PSOBOA algorithm is obviously better than that of PSO, CSA, BOA and BBA, and the optimization stability is the best. The above analysis shows that the overall optimization ability of PSOBOA is better than that of PSO, CSA, BOA, and BBA.

### 6.2. Simulation Environment Construction

The proposed algorithm was tested using the database’s standard classification data set, and a number of comparisons and experiments were conducted with conventional PSO-KELM [40], BBA-KELM [41], BOA-KELM, and other algorithms in order to confirm its viability and effectiveness. Windows 10 (64-bit), MATLAB 2020b, a 12th Gen Intel(R) Core(TM) i9-12900 CPU running at 3.20 GHz, and 32G of RAM were used as the simulation experiment setting.

We evaluated the classification performance of this method, which are classification accuracy (ACC), sensitivity (SEN), specificity (SPE), precision(PRE), and F-measure, which are defined as follows:

Accuracy is the proportion of the total number of correct predictions. Use the following methods to determine it:

(24) $$ACC=\frac{TP+TN}{TP+TN+FN+FP}\times 100\%$$

Sensitivity is an index used to measure the classifier’s recognition of abnormal records, and is also often expressed as the *TP* rate.

(25) $$SEN=\frac{TP}{TP+FN}\times 100\%$$

Specificity is often used to estimate the ability of a classification model to identify normal examples, which is also often expressed as the *TN* rate.

(26) $$SPE=\frac{TN}{TN+FP}\times 100\%$$

Precision is the correct proportion of positive instances of prediction, calculated using:

(27) $$PRE=\frac{TP}{TP+FP}\times 100\%$$

Among them, *TP*, *FP*, *TN*, and *FN* represent true positive, false positive, true negative, and false positive, respectively.

Lewis and Gale proposed the *F*-measure in 1994, which is defined as follows:

(28) $$F-=\frac{(\beta^{2}+1)\times \mathrm{Pr}ecision\times Sensitivity}{\beta^{2}\times \mathrm{Pr}ecision+Sensitivity}$$

In Equation (28), there is a value from 0 to infinity to control the weights assigned to the precision and sensitivity. If all positive instances are classified incorrectly, any classifier evaluated using the above will have a metric of 0. In this experiment, the β value was set to 1.

### 6.3. Algorithm Test Comparison and Result Analysis

In order to verify the effectiveness of the proposed method, this part experiments on the PSOBOA-KELM algorithm on seven classification data sets, which are BreastEW, CongressEW, Hepatitis, JPNdata, Parkinson, SpectEW, and Wdbc. The data sets are from the UCI Machine Learning Library (http://archive.ics.uci.edu/mL/datasets, accessed on 1 October 2022). These data sets are mainly divided into binary classification problems, multi-classification problems, and regression fitting problems. The Breastcancer dataset has 699 data, including 9 features and two categories; the Parkinson dataset has 195 data, including 23 features and two categories; the BreastEW dataset has 569 data, including 30 features and two categories; and the Dermatology dataset has 358 data, including 35 features and six categories. The experiments selected seven real datasets widely used for multi-label classification. The learning factor (*c*_1_ = *c*_2_ = 2) in the important parameters of the particle swarm optimization algorithm was the inertia weight factor *w*_1_ = 0.9 and *w*_2_ = 0.4. Table 2 summarizes the data size, attribute dimension, number of tags, and cardinality of the seven datasets. The specific description information of the dataset is shown in Table 2.

The data set had to be preprocessed before the experiment, and certain features were missing. These records were averaged in this experiment to guarantee the accuracy of the sample data. To reduce the gap between the eigenvalues and prevent the larger eigenvalues from adversely affecting the smaller eigenvalues, we normalized each eigenvalue to the [−1, 1] interval. The normalized calculation formula is:

(29) $$x^{\prime }=\left(\frac{x-\min_{a}}{\max_{a}-\min_{a}}\right)*2-1$$

where *x* is the original value of the data, $x^{\prime }$ is the normalized value, $\max_{a}$ is the maximum value in feature *a*, and $\max_{a}$ is the minimum value in feature *a*.

At the same time, in order to obtain an unbiased estimate of the algorithm’s generalization accuracy, *k*-fold CV is generally used to evaluate the classification accuracy. In this method, all test sets are independent, which can improve the reliability of the results. In this study, the *k* value was set to 10, that is, each experimental data set was divided into 10 subsets, one of which was taken as the test set each time, and the rest was used as the training set, and then the average value of 10 experiments was calculated as the result of the ten-fold crossover. Each of the above classification experiments was run independently 20 times to ensure the stability of the algorithm.

The parameter settings of the contrast swarm intelligent optimization algorithm involved in this paper are shown in Table 3.

From the results of Table 4 and Table 5, it can be seen that the method proposed in this paper is accurate in accuracy, precision, F-measure, sensitivity, specificity, and MCC. The indicator performs significantly better than other comparative feature selection methods.

For the accuracy indicator, the PSOBOA-KELM feature selection method proposed in this paper has an accuracy rate of 96.49%, 96.56%, 87.87%, 83.96%, and 90% on BreastEW, CongressEW, hepatitisfulldata, JPNdata, Parkinson, SpectEW, and wdbc data sets, respectively. Compared with the PSO-KELM, BBA-KELM, and BOA-KELM feature selection methods, the method proposed in this paper has the highest accuracy rate. For example, in the BreastEW dataset, the accuracy of the method proposed in this paper is 0.91% higher than the PSO-KELM method, 0.91% higher than the BBA-KELM method, and 0.03% higher than the BOA-KELM method. For the precision indicator, on the BreastEW, CongressEW, hepatitisfulldata, JPNdata, Parkinson, SpectEW, and wdbc data sets, the accuracy of the PSOBOA-KELM feature selection method proposed in this paper is 95.98%, 100%, 100%, 78.89%, 92.86%, 87.5%, and 100%, respectively. Compared with the PSO-KELM, BBA-KELM, and BOA-KELM feature selection methods, the proposed method has the highest accuracy. For example, in the BreastEW dataset, the accuracy of the method proposed in this paper is 1.31% higher than the PSO-KELM method, 1.46% higher than the BBA-KELM method, and 1.31% higher than the BOA-KELM method. For the F-measure index, on the BreastEW, CongressEW, hepatitisfulldata, JPNdata, Parkinson, SpectEW, and wdbc data sets, the F-measure of the PSOBOA-KELM feature selection method proposed in this paper is 97.3%, 97.10%, 70.83%, 84.03%, 93.75%, 33.33%, and 96.4%, respectively. Compared with the PSO-KELM, BBA-KELM, and BOA-KELM feature selection methods, the proposed method’s F-measure works better. For example, in the CongressEW dataset, the F-measure value of the method proposed in this paper is 2.76% higher than the PSO-KELM method, 4.79% higher than the BBA-KELM method, and 0.95% higher than the BOA-KELM method.

For the sensitivity index, on the BreastEW, CongressEW, hepatitisfulldata, JPNdata, Parkinson, SpectEW, and wdbc data sets, the sensitivity values of the PSOBOA-KELM feature selection method proposed in this paper are 100%, 94.37%, 58.33%, 93.75%, 100%, 20%, and 93.07%, respectively. Compared with the PSO-KELM, BBA-KELM, and BOA-KELM feature selection methods, the proposed method has a higher sensitivity value. For example, in the CongressEW dataset, the sensitivity value of the method proposed in this paper is 1.92% higher than the PSO-KELM method, 1.78% higher than the BBA-KELM method, and 1.78% higher than the BOA-KELM method. Compared with the PSO-KELM, BBA-KELM, and BOA-KELM feature selection methods, the method proposed in this paper has a higher specificity value. For example, in the BreastEW dataset, the specificity value of the method proposed in this paper is 1.96% higher than the PSO-KELM method, 2.38% higher than the BBA-KELM method, and 2.38% higher than the BOA-KELM method. For the MCC index, the PSOBOA-KELM feature selection method proposed in this paper has the MCC values of 92.58%, 93.16%, 66.39%, 68.1%, and 72.81% on the BreastEW, CongressEW, hepatitisfulldata, JPNdata, Parkinson, SpectEW, and wdbc data sets, respectively. Compared with the PSO-KELM, BBA-KELM, and BOA-KELM feature selection methods, the method proposed in this paper has a higher MCC value. For example, in the wdbc data set, the MCC value of the method proposed in this paper is 3.64% higher than the PSO-KELM method, 5.61% higher than the BBA-KELM method, and 1.93% higher than the BOA-KELM method.

**Figure 3:**
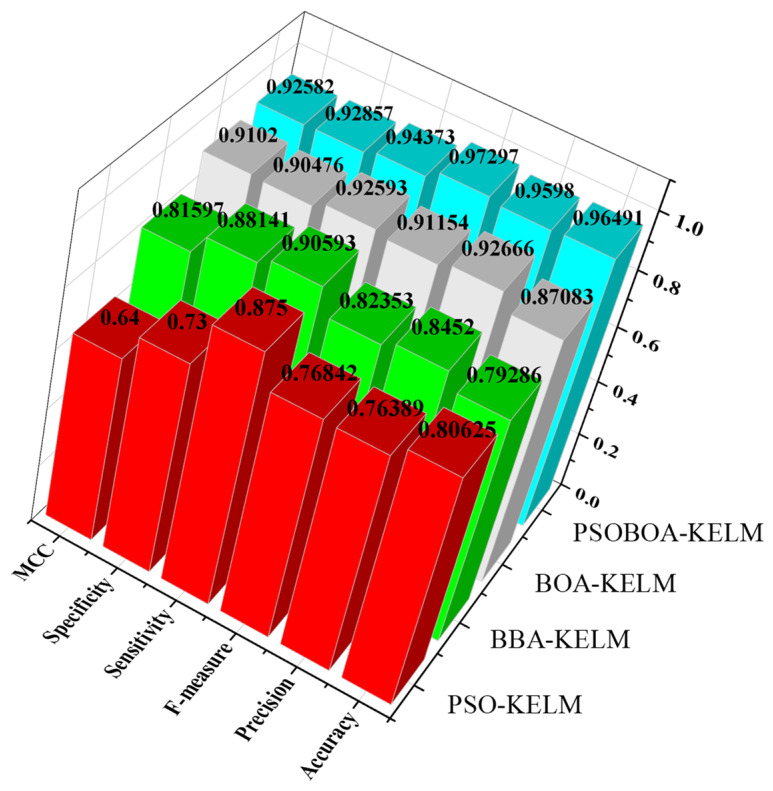
Comparison of the evaluation index parameters of the four algorithms.

**Figure 4:**
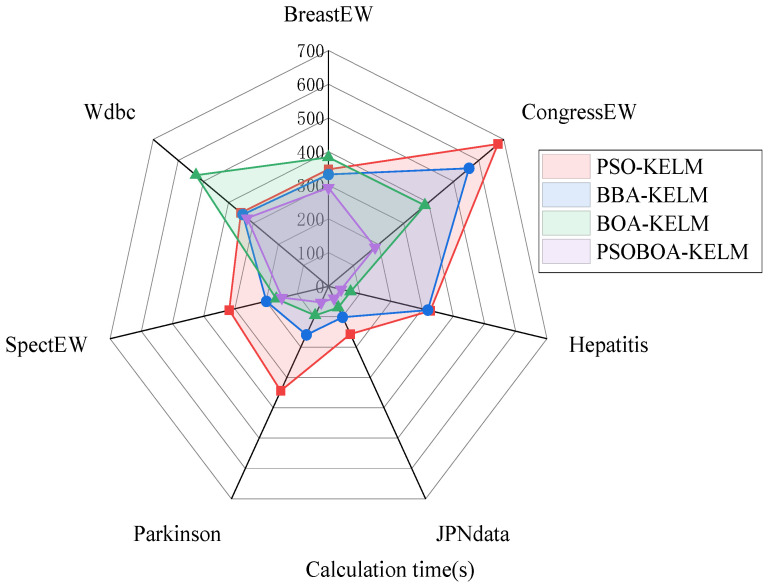
Comparison of the simulation time consumption of the four algorithms.

In addition, in order to compare the performance of these four algorithms more intuitively, as shown in Figure 3, the performance evaluation indicators of these four methods are compared in detail. At the same time, the calculation and simulation time consumption of the four algorithms in the seven data sets are also presented, as shown in Figure 4.

**Figure 5:**
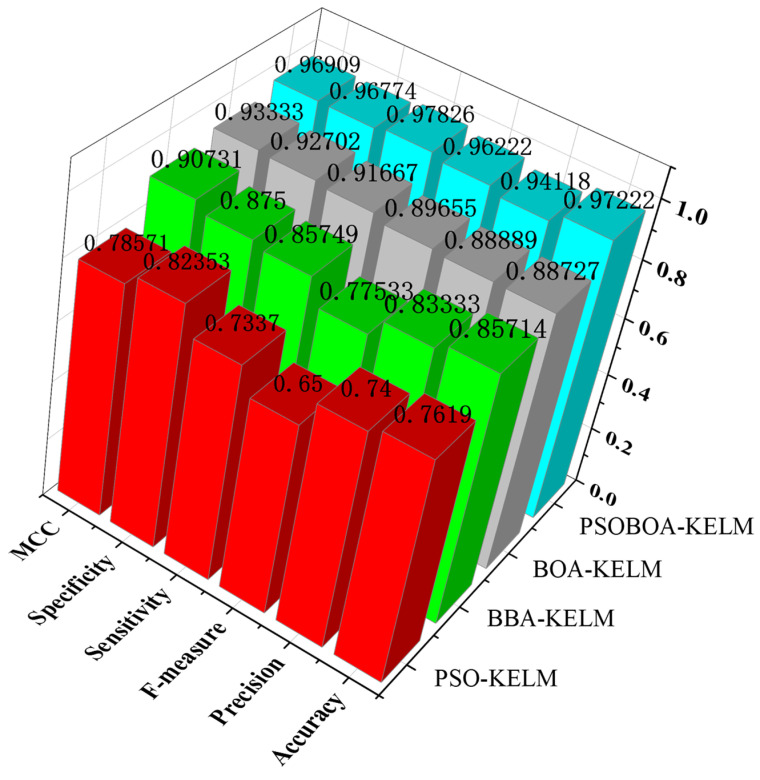
Comparison of the evaluation index parameters of the four algorithms.

**Figure 6:**
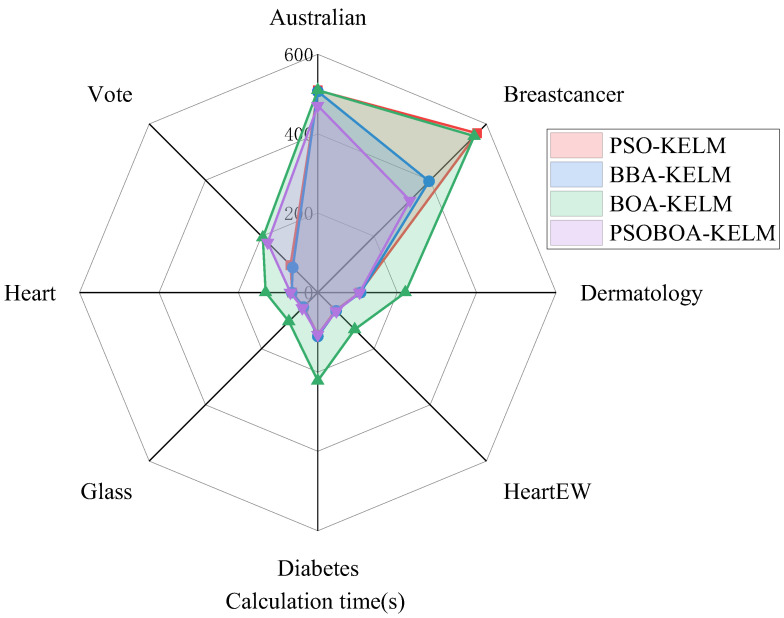
Comparison of the simulation time consumption of the four algorithms.

According to the experimental findings, the PSOBOA-KELM technique has an acceptable performance in terms of classification, and the calculation and simulation times are not too long. It may choose an acceptable and constrained feature subset, and its classification performance is noticeably better than that of comparable approaches. The algorithm also performs well when it comes to the challenge of classifying various data sets. It takes a fair amount of time and produces an excellent classification accuracy. The comparison of eight datasets is shown in Table 6 and Table 7. In addition, in order to compare the performance of these four algorithms more intuitively, the performance evaluation indicators of these four methods are compared in detail, as shown in Figure 5. At the same time, the calculation and simulation time consumption of the four algorithms in the seven data sets are also presented, as shown in Figure 6.

The four algorithms were tested in the Australian, Breastcancer, Dermatology, HeartEW, Diabetes, Glass, Heart, and Vote8 data sets regarding accuracy, precision, F-measure, sensitivity, specificity, MCC, and other six indicators, and achieved a good classification performance. The calculation and simulation time were also relatively short for the PSOBOA-KELM method. It may choose an acceptable and constrained feature subset, and its classification performance is noticeably better than that of comparable approaches. In addition to achieving an improved classification accuracy, the algorithm also performs well while classifying data from various data sets.

In addition, the simulation experiment comparison of the Sinc function was conducted. The four algorithms were compared by fitting the Sinc function. The expression of the Sinc function is as follows:

(30) $$f(x)=\left\{ \begin{array}{ll} \frac{\sin(x)}{x}, & x\neq 0\\ 0\;, & x=0\; \end{array}\right.$$

We set to generate 2000 [−10, 10] uniformly distributed data sets *x*, calculated 2000 data sets $\left\{x_{i},f(x_{i})\right\},i=1,2,3,\ldots ,2000$, and then generated 2000 [−0.2, 0.2] uniformly distributed noise *ε*. Let the training set be $\left\{x_{i},f(x_{i})+\varepsilon_{i}\right\},i=1,2,3,\ldots ,2000$ and then generate another set of 2000 data sets $\left\{y_{i},f(y_{i})\right\},i=1,2,3,\ldots ,2000$ as the test set. In addition, the root-mean-square error (*RMSE*), mean absolute error (*MAE*), and relative standard deviation (*RSD*) were used as the evaluation indicators for error analysis. The calculation formulas of the three indicators are as follows:

(31) $$RMSE=\sqrt{\frac{1}{N}\sum_{i=1}^{N}{\left(y(i)-y^{\prime }(i)\right)}^{2}}$$

(32) $$MAE=\frac{1}{N}\sum_{i=1}^{N}\left|y^{\prime }(i)-y(i)\right|=\frac{1}{N}\sum_{i=1}^{N}\left|e_{i}\right|$$

(33) $$RSD=\frac{\sqrt{\sum_{i=1}^{N}{\left(y^{\prime }(i)-{\bar{y}}^{\prime }(i)\right)}^{2}}}{\sqrt{\sum_{i=1}^{N}{\left(y(i)-{\bar{y}}^{\prime }(i)\right)}^{2}}}$$

where parameter y(i) represents the measured value, $y^{\prime }(i)$ represents the predicted value, parameter *N* is the number of samples, parameter $e_{i}$=$y^{\prime }(i)-y(i)$ is the absolute error, and the numerator and denominator of RSD are in the form of standard deviation. The comparison of Sinc function fitting results is shown in Table 8.

It can be seen from Table 8 that, calculated by the PSO-KELM algorithm, the index values of RMSE and MAE are the largest, and the index value of RSD is closer to the smallest, and the performance of the test results is poor. The index value is even smaller, and the performance of the test result is average. The RMSE and MAE index values of the BOA-KELM algorithm are smaller, the RSD index values are closer to larger, and the test results have a better performance. The PSOBOA-KELM algorithm has the smallest RMSE and MAE index values, the RSD index value is closer to the largest, and the test results have the best performance. It shows that the error of the PSOBOA-KELM model is relatively smaller, and the prediction accuracy is better than that of the PSO-KELM, BBA-KELM, and BOA-KELM algorithms. At the same time, this can also be known from the data change trend in Table 2, which indicates that the PSOBOA-KELM algorithm has the best performance, and optimizing the KELM regularization parameter *C* and kernel function *S* can improve the prediction accuracy of the KELM model.

## 7. Conclusions

The model selection problem of kernel extreme learning machines was investigated in this paper utilizing an enhanced butterfly technique that is based on particle swarm optimization (PSOBOA). This study compared the PSO-KELM, BBA-KELM, and BOA-KELM approaches in-depth to the proposed PSOBOA-KELM model. To assess the model’s performance, we used six indicators: accuracy, precision, F-measure, sensitivity, specificity, and MCC. According to the experimental findings, PSOBOA-KELM can swiftly converge to the best solution inside the search space. The model may combine the benefits of the PSOBOA and KELM models and has a good optimization performance thanks to the inclusion of the original butterfly optimization method in the particle swarm search approach. Better performance, fewer algorithm parameters, and quick search times are some of its features. The performance as well as the classification accuracy have both dramatically increased.

Research on the issue of vast sample sparsity in a high-dimensional space will be the next stage, followed by the in-depth study of the categorization of large sample data by logically making use of useful historical data.

## Figures and Tables

**Table 1:** Test functions.

Function	Equation	Dimension	Bounds	Optimum
F1	${\sum_{i=1}^{d}\left(\sum_{j=1}^{i}x_{j}\right)}^{2}$	30	[−100, 100]	0
F2	$\max\left\{\left|x_{i}\right|,1\leq i\leq d\right\}$	30	[−100, 100]	0
F3	$\sum_{i=1}^{d}\left|x_{i}\sin(x_{i})+0.1x_{i}\right|$	30	[−10, 100]	0
F4	$-20\exp\left(-0.2\sqrt{\frac{1}{d}\sum_{i=1}^{d}x^{2}}\right)-\exp\left(\frac{1}{d}\sum_{i=1}^{d}\cos(2\pi x_{i})\right)+20+\exp(1)$	30	[−5.12, 5.12]	0
F5	$-\sum_{i=1}^{4}c_{i}\exp\left(-\sum_{j=1}^{3}a_{ij}{\left(x_{j}-p_{ij}\right)}^{2}\right)$	30	[1, 3]	−3.86
F6	$-\sum_{i=1}^{7}{\left[(X-a_{i}){\left(X-a_{i}\right)}^{T}+c_{i}\right]}^{-1}$	30	[0, 10]	−10.4
F7	$-\sum_{i=1}^{10}{\left[(X-a_{i}){\left(X-a_{i}\right)}^{T}+c_{i}\right]}^{-1}$	30	[0, 10]	−10.5
F8	$-\sum_{i=1}^{5}{\left[(X-a_{i}){\left(X-a_{i}\right)}^{T}+c_{i}\right]}^{-1}$	30	[0, 10]	−10.1

**Table 2:** Detailed description of the dataset.

Number	Data Set	Number of Categories	Sample Size	Number of Features
1	BreastEW	2	569	9
2	CongressEW	2	435	16
3	Hepatitis	2	1385	29
4	JPNdata	4	148	18
5	Parkinson	2	197	23
6	SpectEW	2	267	22
7	wdbc	2	569	30

**Table 3:** Parameter settings of the swarm intelligence optimization algorithm.

Algorithm	Parameters
PSO	*V_max_* = 4, *w_Max_* = 1, *w_Min_* = 0.9, *c*_1_ = 1.5, *c*_2_ = 2.
BBA	$\left[f_{\min},f_{\max}]=[0,2\right]$; A=0.5; r=0.5; α=0.9; γ=0.05
BOA	*p* = 0.8, power_exponent = 0.1, sensory_modality = 0.01.
PSOBOA	*p* = 0.8, power_exponent = 0.1, sensory_modality = 0.01. *w_Max_* = 0.2, *w_Min_* = 0.9.

**Table 4:** Experimental results of four datasets: BreastEW, CongressEW, Hepatitis, and JPNdata.

Metrics	Algorithm	BreastEW	CongressEW	Hepatitis	JPNdata
Accuracy	PSO-KELM	0.95583	0.93182	0.80625	0.80625
	BBA-KELM	0.95583	0.90909	0.87083	0.74167
	BOA-KELM	0.9646	0.95455	0.87083	0.79286
	PSOBOA-KELM	0.96491	0.96564	0.87868	0.83958
precision	PSO-KELM	0.94666	1	0.5	0.76389
	BBA-KELM	0.9452	0.96	1	0.77778
	BOA-KELM	0.94666	1	0.875	0.77778
	PSOBOA-KELM	0.9598	1	1	0.78889
F-measure	PSO-KELM	0.96517	0.9434	0.57143	0.82353
	BBA-KELM	0.96611	0.92308	0.5	0.76842
	BOA-KELM	0.9726	0.96154	0.66667	0.80065
	PSOBOA-KELM	0.97297	0.97097	0.70833	0.84034
Sensitivity	PSO-KELM	0.98611	0.9245	0.66667	0.875
	BBA-KELM	1	0.92593	0.33333	0.86607
	BOA-KELM	1	0.92593	0.58333	0.875
	PSOBOA-KELM	1	0.94373	0.58333	0.9375
Specificity	PSO-KELM	0.90909	1	0.88141	0.73214
	BBA-KELM	0.90476	0.94118	1	0.71429
	BOA-KELM	0.90476	1	1	0.75
	PSOBOA-KELM	0.92857	1	1	0.75
MCC	PSO-KELM	0.90622	0.86923	0.45227	0.64569
	BBA-KELM	0.90731	0.81597	0.536	0.57071
	BOA-KELM	0.92547	0.9102	0.61899	0.58872
	PSOBOA-KELM	0.92582	0.93158	0.66387	0.68104

**Table 5:** Experimental results of three datasets: Parkinson, SpectEW, and Wdbc.

Metrics	Algorithm	Parkinson	SpectEW	Wdbc
Accuracy	PSO-KELM	0.84605	0.81456	0.95659
	BBA-KELM	0.82105	0.7967	0.9469
	BOA-KELM	0.875	0.81481	0.96491
	PSOBOA-KELM	0.9	0.84615	0.97383
precision	PSO-KELM	0.89904	0.58333	1
	BBA-KELM	0.83333	0	1
	BOA-KELM	0.87451	0.63333	1
	PSOBOA-KELM	0.92857	0.875	1
F-measure	PSO-KELM	0.89606	0.36508	0.93841
	BBA-KELM	0.87778	0	0.92308
	BOA-KELM	0.9233	0.39286	0.95
	PSOBOA-KELM	0.9375	0.33333	0.964
Sensitivity	PSO-KELM	0.93095	0.26667	0.8842
	BBA-KELM	0.92857	0	0.85714
	BOA-KELM	1	0.28333	0.90476
	PSOBOA-KELM	1	0.2	0.93074
Specificity	PSO-KELM	0.675	0.95346	1
	BBA-KELM	0.4	1	1
	BOA-KELM	0.6	1	1
	PSOBOA-KELM	0.75	1	1
MCC	PSO-KELM	0.59994	0.30364	0.90873
	BBA-KELM	0.46155	0	0.88901
	BOA-KELM	0.65248	0.40005	0.92582
	PSOBOA-KELM	0.72809	0.40988	0.94514

**Table 6:** Experimental results of the four datasets of Australian, Breastcancer, Dermatology, and HeartEW.

Metrics	Algorithm	Australian	Breastcancer	Dermatology	HeartEW
Accuracy	PSO-KELM	0.83333	0.85749	0.77533	0.85185
	BBA-KELM	0.95583	0.90909	0.87083	0.74074
	BOA-KELM	0.92702	0.9375	0.91667	0.85185
	PSOBOA-KELM	0.94429	0.98571	0.97297	0.92593
precision	PSO-KELM	0.74074	0.73333	0.78571	0.70314
	BBA-KELM	0.85185	0.83333	0.84615	0.77778
	BOA-KELM	0.92593	0.83871	0.875	0.88889
	PSOBOA-KELM	0.94118	0.97826	0.94444	0.92857
F-measure	PSO-KELM	0.66667	0.77778	0.63246	0.65327
	BBA-KELM	0.85749	0.83333	0.77533	0.77778
	BOA-KELM	0.86667	0.91667	0.92857	0.89655
	PSOBOA-KELM	0.92308	0.98901	0.97222	0.9375
Sensitivity	PSO-KELM	0.7337	0.77778	0.70314	0.8325
	BBA-KELM	0.82746	0.83333	0.85749	0.87607
	BOA-KELM	0.88235	0.96296	0.91667	0.90909
	PSOBOA-KELM	0.92308	0.97826	0.94444	0.93427
Specificity	PSO-KELM	0.79057	0.77533	0.83333	0.82353
	BBA-KELM	0.875	0.85749	0.85185	0.92857
	BOA-KELM	0.92702	0.91667	0.92593	0.92702
	PSOBOA-KELM	0.96774	0.95842	0.94118	0.93667
MCC	PSO-KELM	0.78571	0.86923	0.70314	0.64569
	BBA-KELM	0.90731	0.90323	0.86667	0.7532
	BOA-KELM	0.88235	0.93333	0.88889	0.84615
	PSOBOA-KELM	0.90889	0.96909	0.95366	0.85749

**Table 7:** Experimental results tested in the Diabetes, Glass, Heart, and Vote data sets.

Metrics	Algorithm	Diabetes	Glass	Heart	Vote
Accuracy	PSO-KELM	0.81571	0.7619	0.66667	0.68182
	BBA-KELM	0.88889	0.85714	0.77273	0.72127
	BOA-KELM	0.91667	0.88727	0.81905	0.80952
	PSOBOA-KELM	0.94444	0.97222	0.87868	0.88958
Precision	PSO-KELM	0.65217	0.69565	0.71429	0.72853
	BBA-KELM	0.72727	0.78381	0.85714	0.83636
	BOA-KELM	0.86273	0.89667	0.8852	0.87273
	PSOBOA-KELM	0.9219	0.95182	0.94182	0.91952
F-measure	PSO-KELM	0.71905	0.75429	0.73913	0.80952
	BBA-KELM	0.77905	0.80953	0.79273	0.86667
	BOA-KELM	0.8895	0.87818	0.89545	0.89143
	PSOBOA-KELM	0.94545	0.96667	0.97429	0.97381
Sensitivity	PSO-KELM	0.68182	0.77533	0.85749	0.83333
	BBA-KELM	0.77902	0.83333	0.87902	0.90235
	BOA-KELM	0.91667	0.92593	0.9375	0.92702
	PSOBOA-KELM	0.95749	0.97373	0.96189	0.98774
Specificity	PSO-KELM	0.66667	0.77533	0.59333	0.78571
	BBA-KELM	0.73333	0.79057	0.75862	0.85743
	BOA-KELM	0.88235	0.91667	0.90819	0.91751
	PSOBOA-KELM	0.96296	0.94323	0.9775	0.96774
MCC	PSO-KELM	0.77533	0.81363	0.70314	0.78235
	BBA-KELM	0.8523	0.88235	0.85749	0.88889
	BOA-KELM	0.90667	0.91321	0.81279	0.92702
	PSOBOA-KELM	0.93333	0.96774	0.97296	0.98774

**Table 8:** Comparison of the Sinc function fitting results.

	PSO-KELM			BBA-KELM			BOA-KELM			PSOBOA-KELM		
Iteration	RMSE	MAE	RSD	RMSE	MAE	RSD	RMSE	MAE	RSD	RMSE	MAE	RSD
5	0.027	0.024	0.915	0.021	0.016	0.929	0.017	0.015	0.945	0.014	0.012	0.963
10	0.025	0.023	0.928	0.019	0.014	0.935	0.015	0.013	0.951	0.012	0.008	0.969
15	0.021	0.021	0.935	0.018	0.013	0.942	0.013	0.012	0.957	0.011	0.007	0.973
20	0.019	0.019	0.941	0.016	0.012	0.949	0.012	0.011	0.963	0.01	0.006	0.98
25	0.018	0.017	0.945	0.015	0.011	0.953	0.011	0.01	0.967	0.01	0.006	0.984
30	0.015	0.015	0.949	0.013	0.01	0.958	0.01	0.009	0.971	0.009	0.005	0.988
35	0.013	0.014	0.953	0.011	0.009	0.962	0.009	0.008	0.975	0.009	0.005	0.99
40	0.012	0.013	0.958	0.01	0.009	0.964	0.009	0.008	0.978	0.007	0.005	0.991
45	0.012	0.011	0.961	0.009	0.009	0.968	0.008	0.007	0.981	0.006	0.004	0.992
50	0.012	0.011	0.961	0.009	0.009	0.971	0.007	0.006	0.983	0.006	0.004	0.993

## Data Availability

The data used to support the findings of this study are available from the corresponding author upon request.
